# Er:YAG Laser-Assisted Versus Conventional Surgery for Medication-Related Osteonecrosis of the Jaw: A Systematic Review and Critical Appraisal of Randomised Evidence

**DOI:** 10.7759/cureus.117540

**Published:** 2026-10-06

**Authors:** Mohamed Xamza, Jaylani Xamza, Abbas Hussein, Mohamed Baalawi, Nora Arbe, Raees Samli, Abdelmadjid Zinet

**Affiliations:** 1 General Surgery, Homerton University Hospital NHS Foundation Trust, London, GBR; 2 Dentistry, Queen Mary University of London, London, GBR; 3 Intensive Care Unit, North Middlesex University Hospital NHS Trust, London, GBR; 4 Emergency Medicine, Barts Health NHS Trust, London, GBR; 5 Neurology, North Middlesex Hospital, London, GBR; 6 Intensive Care Unit, Royal Blackburn Teaching Hospital, Blackburn, GBR; 7 Orthopaedics and Trauma, Northwick Park Hospital, London, GBR

**Keywords:** conventional surgery, er:yag laser, laser-assisted surgery, medication-related osteonecrosis, randomized controlled trial, systematic review

## Abstract

Medication-related osteonecrosis of the jaw (MRONJ) is a serious complication of antiresorptive and antiangiogenic therapy. Er:YAG (erbium-doped yttrium aluminium garnet) laser-assisted surgery has been proposed as an adjunct to conventional debridement, but randomised evidence is limited.

This PROSPERO-registered systematic review (CRD420261509183), conducted according to PRISMA 2020, searched PubMed/MEDLINE, Embase, Scopus, and the Cochrane Library through September 10, 2026 for randomised studies comparing laser-assisted surgery with conventional surgical treatment of MRONJ. Thirteen full-text articles were assessed, and one randomised controlled trial met eligibility criteria. The trial report contains inconsistent participant accounting; its methods and results state that 72 patients were randomised and 65 completed follow-up, whereas the abstract and trial registry report 65 participants. No meta-analysis was performed. In complete-case analyses over 24 weeks of follow-up, adjunctive Er:YAG laser surgery was associated with higher short-term complete mucosal healing (85.7% vs 26.7%; review-calculated risk ratio (RR) 3.21, 95% CI 1.75-5.91), fewer postoperative complications (17.1% vs 60.0%; RR 0.29, 95% CI 0.13-0.63), and lower pain scores at three and six months, with longer procedure duration. Extreme-case sensitivity analyses for seven patients without reported healing outcomes did not change the direction of effect. Risk of bias raised some concerns for all outcomes. GRADE (Grading of Recommendations Assessment, Development, and Evaluation) certainty was low for healing, complications, and pain, very low for necrosis, and moderate for procedure duration. These apparent benefits derive from a single small trial, with low to very low certainty for all clinical outcomes, and apply only to adjunctive Er:YAG ablation in stage II-III MRONJ. They should not be extrapolated to photobiomodulation, other laser modalities, or other disease stages. Independent randomised evidence is required before its effectiveness can be established.

## Introduction and background

Introduction

Medication-related osteonecrosis of the jaw (MRONJ) is a serious adverse effect associated most commonly with antiresorptive therapy, including bisphosphonates and denosumab, and has also been reported with antiangiogenic and other targeted therapies [1-6]. The 2022 American Association of Oral and Maxillofacial Surgeons (AAOMS) case definition requires all three of the following: current or previous antiresorptive therapy, alone or in combination with immune modulators or antiangiogenic medications; exposed bone or bone probeable through an intraoral or extraoral fistula in the maxillofacial region persisting for more than eight weeks; and no history of radiation therapy to the jaws or metastatic disease to the jaws [6]. MRONJ is multifactorial, with impaired bone remodelling, infection or inflammation, and altered soft-tissue and vascular healing among the proposed mechanisms [2,6]. Clinical presentation varies widely, from asymptomatic exposed bone to pain, infection, fistula, and pathologic fracture, and risk differs by medication type, dose, and indication, being substantially higher with high-dose antiresorptive therapy for malignancy than with treatment for osteoporosis [5,6].

The AAOMS staging system ranges from stage 0, in which there is no clinically exposed necrotic bone, to stage III, which may include extension beyond alveolar bone, pathologic fracture, extraoral fistula, oroantral or oronasal communication, or osteolysis to the mandibular border or sinus floor [6]. Management has moved away from reserving surgery only for advanced disease [5,6]. Nonoperative therapy, including antimicrobial mouth rinses, systemic antibiotics, analgesia, and patient education, remains an option in the 2022 AAOMS update, and antibiotic prophylaxis has also been proposed around invasive dental procedures in at-risk patients [6,7]. However, the update states that operative therapy is increasingly supported for all stages, including stage I, and recommends timely surgery when nonoperative therapy fails or when disease is progressive or advanced at presentation [6,8,9]. This shift has increased interest in techniques that might improve debridement while minimising additional tissue injury [10].

Conventional surgical debridement commonly uses rotary instruments under irrigation [10]. Er:YAG (erbium-doped yttrium aluminium garnet) laser surgery uses a 2,940-nm wavelength with strong absorption by water and hydroxyapatite, allowing hard-tissue ablation with limited thermal spread and without the friction of rotary cutting [11-14]. Proposed advantages include removal of the smear layer and local decontamination of the treated bone surface [13-15]. These effects should be distinguished from photobiomodulation or low-level laser therapy, which uses different biological mechanisms, doses, and treatment goals [14,16,17]. Accordingly, evidence for Er:YAG surgical ablation should not be generalised to photobiomodulation, Nd:YAG (neodymium-doped yttrium aluminium garnet), CO2, or diode protocols without direct comparative data.

Earlier reviews were based largely on nonrandomised evidence. Momesso et al. [18] included 15 studies published from 2006 to 2018, comprising 10 retrospective studies, 1 prospective study, and 4 case series; only 3 contributed to their meta-analysis. Li et al. [14] included 10 studies across retrospective, prospective, nonrandomised, and other clinical designs and concluded that although laser-assisted approaches appeared promising, the evidence was insufficient for firm conclusions and higher-quality randomised studies were needed. Hanna et al. [17] focused specifically on photobiomodulation rather than ablative Er:YAG surgery; 12 of 126 screened studies were eligible, methodological and outcome heterogeneity precluded meta-analysis, and most included evidence was at high risk of bias. The 2022 Cochrane review [19] of MRONJ interventions included 13 randomised trials with 1,668 participants across diverse prevention and treatment strategies. Because the interventions were clinically heterogeneous, meta-analysis was not possible, and certainty was generally low or very low.

Previous reviews therefore could not establish whether laser-assisted surgery improves outcomes compared with conventional surgery under randomised allocation because their conclusions rested mainly on retrospective and nonrandomised comparisons that are vulnerable to selection bias and to confounding by disease stage and medication exposure [14,17-19]. The evidence base changed with publication of the first clearly described randomised controlled trial directly comparing conventional surgery plus adjunctive Er:YAG laser with conventional surgery alone for stage II-III MRONJ [15]. Unlike earlier reviews, the present review restricts its primary synthesis to randomised evidence with a documented random sequence-generation method and applies outcome-level risk-of-bias and certainty assessment. Its objectives were to identify all randomised trials comparing laser-assisted with conventional surgery alone for MRONJ; to characterise the effects of laser-assisted surgery on complete mucosal healing, postoperative complications, residual necrosis, pain, and procedure duration; and to appraise the risk of bias and certainty of this evidence. The number of eligible studies was treated as a result of the systematic search rather than as a precondition for undertaking the review [20].

## Review

Methods

Study Design and Registration

This systematic review was conducted in accordance with the Preferred Reporting Items for Systematic Reviews and Meta-Analyses (PRISMA) 2020 guidelines [21] and registered with PROSPERO (registration number CRD420261509183).

Eligibility Criteria

Studies were eligible if they directly compared laser-assisted or laser-adjunctive treatment with conventional surgical treatment alone in patients undergoing surgical management of MRONJ/BRONJ. Eligible laser modalities included Er:YAG, Nd:YAG, CO₂ and diode lasers, as well as photobiomodulation/low-level laser therapy when used as an adjunct to surgical treatment.

Only studies reporting a documented random sequence-generation method, such as computer-generated randomisation or a random-number table, were eligible for the primary randomised-evidence synthesis. Quasi-random methods, such as alternation, date of birth, medical-record number, or admission date, were excluded. Study design was determined from the reported recruitment and allocation methods rather than from database indexing alone. Atalay et al. (2011) [22] illustrate the resulting boundary: its abstract explicitly describes the study as retrospective, while the Methods state that 20 patients were "randomly divided" into two groups, but no sequence-generation or allocation-concealment method is reported. Because the prespecified eligibility standard required a documented random allocation mechanism, this study was excluded from the primary randomised evidence base and is discussed separately as near-eligible comparative evidence (Appendix B, S2).

Studies were excluded if they were case reports or case series without a comparator group, narrative or systematic reviews, conference abstracts without an available full-text publication, or studies evaluating laser therapy for indications other than surgical management of established MRONJ/BRONJ. Studies comparing two active laser or surgical techniques to one another, rather than a laser-assisted approach against conventional surgery alone, were also excluded. Only articles published in English were included, for reasons of translation resource and capacity available to this review team; this restriction is acknowledged as a limitation given the potential for language bias in a very small evidence base.

Primary Outcome

The review was registered in PROSPERO before a single primary outcome was designated. Complete mucosal healing was subsequently selected as the primary outcome because it represents clinical resolution of MRONJ. This constituted a post-registration outcome-prioritisation amendment and is reported transparently. The included trial's registry lists postoperative complications as its primary outcome [23].

Secondary Outcomes

Secondary outcomes included the rate of postoperative complications, postoperative pain (visual analogue scale, assessed at three and six months), the presence of grade II/III necrosis at the three-month assessment (a cross-sectional measure of disease severity at that timepoint, not a relapse-after-healing "recurrence"), and intraoperative duration of the surgical procedure. Preoperative bone-turnover markers (e.g., serum CTX) and baseline disease stage, where reported in the wider non-randomised literature, were treated as prognostic variables or potential effect modifiers rather than as direct treatment-effect outcomes.

Literature Search Strategy

PubMed/MEDLINE, Embase, Scopus, and the Cochrane Library (CENTRAL) were searched from database inception through September 10, 2026. Search concepts combined controlled vocabulary and free-text terms for medication-related or bisphosphonate-related osteonecrosis of the jaw, laser modalities including Er:YAG, Nd:YAG, CO2, diode, and photobiomodulation or low-level laser therapy, surgical treatment, and randomised or controlled trials. A broader sensitivity search omitting study-design terms was also performed because randomised trials may be inconsistently indexed. Reference lists of relevant reviews and the included trial were hand-searched, and forward citation searching was conducted for the included trial. The ClinicalTrials.gov record for the included trial (NCT07029815) [23] was examined for trial-design and risk-of-bias information rather than used as a systematic discovery source. Full electronic search strategies are provided in Appendix A.

Study Selection

Two reviewers independently screened all titles and abstracts and independently assessed full texts against the eligibility criteria. Disagreements were resolved by discussion and consensus, with adjudication by a third reviewer when required. Reasons for full-text exclusion were recorded. The selection process is summarised in Figure 1.

**Figure 1:**
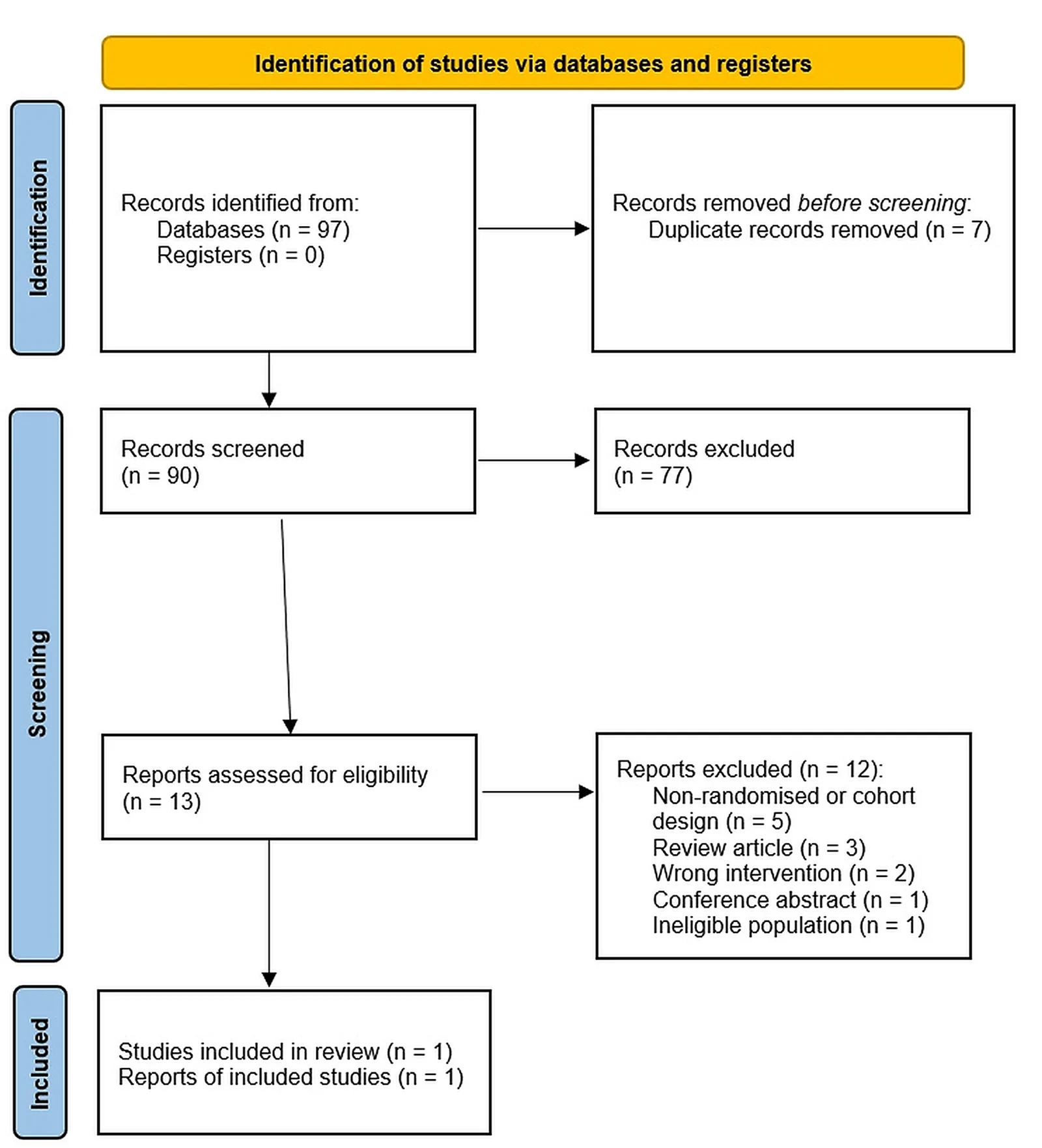
PRISMA 2020 flow diagram of the study selection process PRISMA: Preferred Reporting Items for Systematic Reviews and Meta-Analyses

Data Collection and Management

Two reviewers independently extracted data from the included trials' published full text using a standardised form based on the Cochrane Handbook data-collection template [24]. Extracted items included study design, country, sample size, participant characteristics, MRONJ stage, intervention and comparator details, laser parameters, and follow-up (Table 1) and all prespecified outcomes (Table 2). Where the publication did not report an effect estimate required for interpretation, risk ratios were calculated using the Wald log-risk-ratio method and mean differences using a normal-approximation two-sample formula from the reported event counts, means, standard deviations, and group sizes. These calculated estimates are identified separately from statistics reported by the trial authors. Calculation details are provided in Appendix A.

**Table 1:** Characteristics of the included study MRONJ: medication-related osteonecrosis of the jaw; Er:YAG: erbium-doped yttrium aluminium garnet; rpm: revolutions per minute; µm: micrometre

Characteristic	Details
Study/design/country	Michalak et al. (2026) [15]. Randomised controlled trial, Poland. Publication describes a double-blind design, with the operating surgeon unblinded but patients, postoperative assessor, and data analyst reported as blinded. ClinicalTrials.gov (NCT07029815) [23] lists no masking. Registration was retrospective.
Population	Stage II-III MRONJ in patients receiving antiresorptive or antiangiogenic therapy.
Sample	Publication abstract and registry: 65 participants (35 laser-assisted, 30 conventional). Publication Methods/Results: 72 randomised (36 vs 36), 65 completed/analyzed (35 vs 30). The report states 106 assessed and 41 excluded, which does not reconcile arithmetically with 72 randomised.
Intervention	Conventional debridement plus adjunctive Er:YAG laser (400 mJ, 6 W, 15 Hz, 800-µm tip, 5/8 water cooling).
Comparator	Conventional debridement alone using 8-mm rotary drills (<40,000 rpm) with saline irrigation.
Follow-up	24 weeks, with visits at 1, 2, 4, 12, and 24 weeks.

**Table 2:** Summary of outcomes in the included randomised controlled trial (Michalak et al., 2026) Reference: [15] RR: risk ratio; CI: confidence interval; MD: mean difference; VAS: visual analog scale; Er:YAG: erbium-doped yttrium aluminium garnet

Outcome (definition; assessment timing)	Laser	Control	Effect estimate(s)	P value
Complete mucosal healing; full epithelialization with no exposed bone, assessed by final follow-up (up to 24 weeks) (complete-case, n = 65)	30/35 (85.7%)	8/30 (26.7%)	Trial-reported: OR (non-healing, conventional vs laser) 18.33 (95% BCaCI 4.69-66.00). Review-calculated: RR (healing, laser vs conventional) 3.21 (95% CI 1.75-5.91). Extreme missing-data scenarios: RR 2.14-3.88 (see Results and Appendix A).	< .001
Postoperative complications; the trial's own registered primary outcome, assessed up to 24 weeks (complete-case, n = 65)	6/35 (17.1%)	18/30 (60.0%)	Trial-reported: OR (conventional vs laser) 7.55 (95% BCaCI 2.17-26.54). Review-calculated: RR (laser vs conventional) 0.29 (95% CI 0.13-0.63).	< .001
Grade II/III necrosis present at the 3-month assessment (a cross-sectional disease-severity measure, not a relapse-after-healing "recurrence") (complete-case, n = 65)	1/35 (2.9%)	10/30 (33.3%)	Trial-reported: OR (conventional vs laser) 18.57 (95% BCaCI 1.25-359,804,391.29). Review-calculated: RR (laser vs conventional) 0.09 (95% CI 0.01-0.63); sparse-event estimate.	0.009
Pain (visual analogue scale), assessed at 3 months (complete-case, n = 65)	1.34 ± 1.39	3.93 ± 2.68	Review-calculated: MD -2.59 (95% CI -3.65 to -1.53; normal approximation).	< .05 (exact value not reported)
Pain (visual analogue scale), assessed at 6 months (complete-case, n = 65)	0.60 ± 0.95	3.13 ± 2.52	Review-calculated: MD -2.53 (95% CI -3.49 to -1.57; normal approximation).	< .05 (exact value not reported)
Procedure duration, minutes; intraoperative, recorded at time of surgery (complete-case, n = 65)	70.03 ± 5.95	57.90 ± 7.84	Trial-reported: mean difference +12.13 min (laser longer). Review-calculated MD +12.13 min (95% CI 8.70-15.56; normal approximation).	< .001

Data Synthesis

As only one randomised controlled trial met inclusion criteria, a quantitative meta-analysis was neither possible nor appropriate; results are synthesised narratively. No pooled effect estimate across studies is reported anywhere in this review.

Risk-of-Bias Assessment

Two reviewers independently assessed risk of bias in the included randomised controlled trial using the Cochrane Risk of Bias 2 (RoB 2) tool (August 2019 version, individually randomised parallel-group trials) [25]. Five domains were evaluated separately for complete mucosal healing, postoperative complications, necrosis at three months, pain, and procedure duration: the randomisation process; deviations from intended interventions; missing outcome data; measurement of the outcome; and selection of the reported result. The RoB 2 signalling questions and algorithm were applied to each outcome, and disagreements were resolved by discussion. Overall risk of bias was rated high when at least one domain was high risk, some concerns when no domain was high risk but at least one raised some concerns, and low risk only when all domains were low risk. Outcome-level judgements are summarised in Table 3, with supporting rationale in Appendix A.

**Table 3:** Outcome-level Cochrane RoB 2 assessment for the included randomised controlled trial RoB 2: Risk of Bias 2; D1: bias arising from the randomisation process; D2: bias due to deviations from intended interventions; D3: bias due to missing outcome data; D4: bias in measurement of the outcome; D5: bias in selection of the reported result

RoB 2 domain	Healing	Complication	Necrosis	Pain	Duration
D1. Randomisation process	Low risk	Low risk	Low risk	Low risk	Low risk
D2. Deviations from intended intervention	Low risk	Low risk	Low risk	Low risk	Low risk
D3. Missing outcome data	Some concerns	Some concerns	Some concerns	Some concerns	Some concerns
D4. Measurement of the outcome	Some concerns	Some concerns	Some concerns	Some concerns	Low risk
D5. Selection of the reported result	Some concerns	Some concerns	Some concerns	Some concerns	Some concerns
Overall	Some concerns	Some concerns	Some concerns	Some concerns	Some concerns

Certainty of Evidence

Certainty of evidence was assessed post hoc for each outcome using GRADE, with randomised evidence initially considered high certainty and then evaluated for risk of bias, inconsistency, indirectness, imprecision, and publication bias [26]. Because only one randomised trial was available, between-study inconsistency and publication bias could not be assessed empirically and were not automatically downgraded on that basis alone. Indirectness was judged in relation to the evidence actually available, namely, adjunctive Er:YAG surgery for stage II-III MRONJ. The assessment is exploratory because it was added after protocol registration. Outcome-level judgements and reasons for downgrading are presented in Appendix A.

Results

Study Selection

The database searches identified 97 records, and no records were identified from trial registers. Hand-searching of reference lists and forward citation searching identified no additional records. After removal of 7 duplicates, 90 records were screened by title and abstract, of which 77 were excluded. Thirteen reports were assessed in full text, and one study, the randomised controlled trial by Michalak et al. [15], met all eligibility criteria. The selection process is summarised in Figure 1.

Twelve reports were excluded after full-text assessment: non-randomised or cohort design (n = 5), review articles (n = 3), wrong intervention (n = 2), conference abstract without an available full-text publication (n = 1), and ineligible population (n = 1). Quasi-randomised studies were not eligible. Atalay et al. [22] was among the reports excluded for non-randomised design; it is highlighted as a near-eligible report because its abstract describes the study as retrospective while its Methods state that patients were randomly divided, without a documented random sequence-generation process (Appendix B, S2).

Study Characteristics

Michalak et al. [15] studied patients with stage II-III MRONJ at a single centre in Poland. The participant accounting is internally inconsistent across the report and registry. The abstract states that 65 patients were randomly allocated (35 laser-assisted, 30 conventional). In contrast, the Methods section reports 106 patients assessed and 72 allocated (36 per group) using a software-generated random table with sealed-envelope concealment, while the Results section again states that 72 were randomised and 65 completed the study. The Results section describes six patients missing from the control arm and one from the laser arm. The report also states that 41 of 106 assessed patients were excluded, which arithmetically yields 65 rather than 72. ClinicalTrials.gov reports actual enrollment of 65 [23]. The review therefore uses the 65 participants with published outcome data for effect estimates and treats the seven additional patients described in the Methods and Results as missing in the extreme-case sensitivity analysis. The trial reported no funding and no conflicts of interest. The full characteristics are summarised in Table 1.

Effect estimates are based on the 65 patients with reported outcome data (35 laser-assisted surgery; 30 conventional surgery), unless stated otherwise (Table 2). Risk ratios and mean differences calculated for this review were derived from the trial's published group data and are labelled separately from the trial's reported results.

Follow-up visits were scheduled at 1, 2, 4, 12, and 24 weeks. Pain was reported at three and six months, and grade II/III necrosis at three months. Mucosal healing status was reported as unchanged between three and six months. Postoperative complication counts were reported without a clearly specified assessment timepoint.

Adjunctive Er:YAG laser surgery was associated with higher complete mucosal healing than conventional surgery alone (85.7% vs 26.7%). The trial's reported odds ratio (18.33, 95% BCaCI 4.69-66.00; P < .001) expresses non-healing in the conventional group relative to the laser group; the review-calculated RR for healing with laser versus conventional surgery was 3.21 (95% CI 1.75-5.91) [15].

Sensitivity Analysis for Missing Data

The reported healing analysis included 65 of the 72 patients described as randomised. Two extreme assumptions were examined for the seven patients without reported healing outcomes: one in the laser-assisted surgery group and six in the conventional group. Under the assumption most favourable to laser-assisted surgery, the missing laser-group patient was considered healed and all six missing conventional-group patients unhealed, giving 31/36 versus 8/36 healed (RR 3.88, 95% CI 2.07-7.24). Under the least favourable assumption, the missing laser-group patient was considered unhealed and all six missing conventional-group patients healed, giving 30/36 versus 14/36 healed (RR 2.14, 95% CI 1.39-3.31). These hypothetical analyses do not establish the missing patients' outcomes or remove the risk of bias from incomplete data, but they show that the direction of the healing estimate is robust to these extreme assumptions.

Postoperative complications: The trial's registered primary outcome [23] occurred in 6/35 patients (17.1%) in the laser group and 18/30 (60.0%) in the conventional group. The review-calculated RR was 0.29 (95% CI 0.13-0.63), corresponding to an observed absolute difference of 42.9 percentage points. Because this estimate derives from one small trial with differential attrition, the magnitude should be interpreted cautiously.

At 3 months, grade II/III necrosis was present in 1/35 patients (2.9%) in the laser group and 10/30 (33.3%) in the conventional group. The review-calculated RR was 0.09 (95% CI 0.01-0.63). Although the observed difference favours laser-assisted surgery, only 11 events occurred, so the magnitude of effect is highly uncertain.

Pain scores were comparable between groups preoperatively (laser 7.09 ± 2.06 vs conventional 6.47 ± 2.53; P > .05) but were significantly lower in the laser group at 3 months (1.34 ± 1.39 vs 3.93 ± 2.68; review-calculated mean difference -2.59, 95% CI -3.65 to -1.53) and 6 months (0.60 ± 0.95 vs 3.13 ± 2.52; review-calculated mean difference -2.53, 95% CI -3.49 to -1.57). Laser-assisted procedures took significantly longer than conventional surgery (70.03 ± 5.95 vs 57.90 ± 7.84 minutes; mean difference +12.13 minutes; P < .001).

Randomisation (D1) was rated low risk because the publication reports software-generated allocation and sealed-envelope concealment; the inconsistent participant accounting was treated as a reporting issue rather than evidence that the sequence itself was nonrandom. Deviations from intended interventions (D2) were rated low risk because no material crossovers or protocol deviations were reported. Missing outcome data (D3) raised some concerns: 65 of 72 participants described as randomised in the Methods and Results sections were analysed, with 6 missing from the conventional group and 1 from the laser group. The reported reasons included cancer deterioration and relocation, and their relationship to the outcomes cannot be excluded. Measurement of outcomes (D4) raised some concerns for healing, complications, necrosis, and pain. The publication states that the operating surgeon was unblinded, but patients, the postoperative assessor, and the data analyst were blinded, whereas the registry lists no masking [15,23]. Procedure duration was considered objectively measured. Selection of the reported result (D5) raised some concerns because the trial was registered retrospectively, participant counts differ between the abstract, Methods/Results, and registry, and some outcome timing or definitions are not fully specified. No outcome was judged high risk overall; all were rated as having some concerns.

Certainty of Evidence

The post hoc GRADE assessment rated certainty as low for complete mucosal healing, postoperative complications, and pain; very low for grade II/III necrosis at three months; and moderate for procedure duration. Downgrading reflected the outcome-level risk-of-bias concerns and, for clinical outcomes, imprecision from a single small trial. Necrosis was downgraded further because only 11 events occurred. Inconsistency and publication bias could not be assessed empirically with one eligible trial and were not automatically downgraded for that reason alone. The full Summary of Findings is provided in Appendix A.

Discussion

This review identified one trial meeting the prespecified randomised-evidence eligibility criteria. Over 24 weeks of follow-up, the trial reported substantially higher mucosal healing and fewer complications with adjunctive Er:YAG surgery, together with lower pain scores at 3 and 6 months, at the cost of an approximately 12-minute-longer procedure [15]. The direction of the healing effect was unchanged under extreme assumptions for the seven participants described as randomised but not analysed. These results represent an important change from the evidence available to earlier laser reviews, which consisted predominantly of retrospective studies, prospective cohorts, and case series [14,18], and from the broader 2022 Cochrane review, in which randomised MRONJ treatment evidence remained heterogeneous and generally low or very low certainty [19].

The magnitude of the observed effects should nevertheless be interpreted cautiously. The evidence comes from a single centre and one small trial, outcome analyses use 65 complete cases rather than the 72 participants described as randomised in the Methods and Results sections, and attrition was markedly unbalanced between groups. The report also contains unresolved participant-flow inconsistencies: the abstract and registry report 65 participants, whereas the Methods and Results sections state that 72 were randomised, and the stated screening arithmetic does not reconcile. In addition, the publication describes patient and assessor blinding while the registry lists no masking. These issues do not negate the observed differences but reduce confidence in their exact magnitude and contributed to the GRADE downgrading.

Atalay et al. (2011) [22] provides useful contextual evidence and highlights the difficulty of classifying older reports. Its abstract calls the study retrospective, but the Methods section states that 20 patients were "randomly divided" into laser and conventional groups without describing sequence generation or allocation concealment. Complete healing occurred in 70% of laser-treated patients and 40% of conventionally treated patients, a difference that was not statistically significant (P = .370). The intervention also combined Er:YAG surgery with postoperative Nd:YAG biostimulation, making it clinically different from the 2026 trial. Excluding Atalay from the primary randomised synthesis is therefore based on the review's documented-randomisation criterion, not on an assumption that no allocation process occurred.

The observed direction of effect is biologically plausible, although mechanism alone cannot establish clinical efficacy. Er:YAG energy at 2,940 nm is strongly absorbed by water-containing hard tissue and can ablate bone with limited collateral thermal effects [11-14]. Laser-treated surfaces may also have less smear layer and lower microbial contamination than rotary-cut surfaces [13-15]. These mechanisms are distinct from photobiomodulation, for which Hanna et al. [17] found highly heterogeneous protocols and insufficient evidence to define a standardised regimen. The clinical effect of surgical Er:YAG should therefore be interpreted separately from evidence for low-level laser or photobiomodulation protocols.

Limitations

This review has several important limitations. First, only one trial met the prespecified randomised-evidence criteria, so consistency across independent trials and publication bias could not be assessed. Second, the included trial contains material participant-flow inconsistencies between its Abstract, Methods, Results, and registry, and its primary analyses use complete-case rather than full randomised denominators. Third, Atalay et al. [22] is difficult to classify because it is self-described as retrospective yet states that patients were randomly divided; exclusion was based on the absence of a documented sequence-generation method and should be considered when interpreting the boundary of the evidence base. Fourth, eligibility covered several laser modalities, but the only included randomised trial evaluated adjunctive Er:YAG surgery, so the findings should not be extrapolated to photobiomodulation, Nd:YAG, CO2, or diode protocols. Fifth, complete mucosal healing was selected as the review's primary outcome after PROSPERO registration, and GRADE was also added post hoc; both amendments are reported transparently. Sixth, the included trial was retrospectively registered and its publication and registry disagree on masking and enrollment. Seventh, although PubMed/MEDLINE, Embase, Scopus, and CENTRAL were searched and citation chasing was performed, trial registries were not systematically searched as discovery sources for unpublished or ongoing studies. In addition, follow-up in the included trial was limited to 24 weeks, so the durability of healing and late recurrence could not be assessed. Finally, the review was restricted to English-language publications, which may introduce language bias.

Independent replication is the priority. Future trials should be prospectively registered before enrollment, use transparent participant-flow reporting and intention-to-treat analysis, prespecify clinically meaningful outcome definitions and time points, and describe sequence generation, concealment, and masking in sufficient detail for independent appraisal. Multicenter trials should stratify or report outcomes by MRONJ stage and medication exposure and should distinguish surgical Er:YAG ablation from photobiomodulation or other laser modalities. Longer-term recurrence, quality of life, reoperation, adverse events, treatment burden, and cost-effectiveness should also be assessed.

## Conclusions

This systematic review identified one trial meeting the prespecified randomised-evidence criteria for adjunctive Er:YAG laser surgery versus conventional surgery in MRONJ. In the 65 participants with published outcome data, adjunctive Er:YAG was associated with higher short-term (24-week) mucosal healing, fewer postoperative complications and grade II/III necrosis at 3 months, and lower pain scores at 3 and 6 months, while procedure duration was longer. Extreme-case assumptions for the seven additional participants described as randomised did not reverse the direction of the healing effect.

Confidence in the magnitude of benefit remains limited by the single-centre evidence base, incomplete outcome data, retrospective registration, and unresolved discrepancies in participant accounting and masking. GRADE certainty is low for healing, complications, and pain, very low for necrosis, and moderate for procedure duration. Because these apparent short-term benefits derive from a single small trial and certainty is low to very low for all clinical outcomes, adjunctive Er:YAG surgery should be regarded as promising but unproven. The findings apply specifically to adjunctive Er:YAG ablation in stage II-III MRONJ and should not be extrapolated to photobiomodulation, other laser modalities, or stage I disease. Independent, adequately powered randomised trials with longer follow-up are required before its effectiveness can be established.

## Appendices

Appendix A

Appendix A includes the data availability statement, search strategy, outcome-level RoB 2 rationale, review-calculated effect estimates, extreme-case sensitivity analysis for missing healing outcomes, and summary of findings.

Data Availability Statement

The study-level data used in this review are derived from published reports and the trial registry. Supplementary materials provide the reproducible electronic search strategies, outcome-level risk-of-bias rationale, review-calculated effect estimates, missing-data sensitivity analyses, GRADE certainty assessment, standardised extraction fields, and the rationale for the key near-eligible exclusion. Original database histories, database exports, and the full screening log are retained by the corresponding author and are available on reasonable request.

Table 4 provides the full electronic search strategies. The search date was September 10, 2026. PubMed/MEDLINE, Embase, Scopus, and CENTRAL were searched from database inception with no date restriction. English-language eligibility was applied during study selection. The strategies below document the search concepts used for the review, including an RCT-focused search and a broader sensitivity search without a study-design filter, where applicable.

**Table 4:** Full electronic search strategies

Database	Search strategy	Notes
PubMed/MEDLINE	Primary RCT-focused search: (("Osteonecrosis of the Jaw"[Mesh] OR "Bisphosphonate-Associated Osteonecrosis of the Jaw"[Mesh] OR "medication-related osteonecrosis of the jaw"[tiab] OR MRONJ[tiab] OR "bisphosphonate-related osteonecrosis of the jaw"[tiab] OR BRONJ[tiab] OR "osteonecrosis of the jaw"[tiab]) AND ("Laser Therapy"[Mesh] OR laser*[tiab] OR "Er:YAG"[tiab] OR erbium[tiab] OR "Nd:YAG"[tiab] OR "CO2 laser"[tiab] OR "carbon dioxide laser"[tiab] OR diode[tiab] OR photobiomodulation[tiab] OR "low-level laser"[tiab]) AND (surg*[tiab] OR debridement[tiab] OR curettage[tiab] OR sequestrectomy[tiab] OR treatment[tiab])) AND (random*[tiab] OR trial[tiab] OR controlled[tiab] OR "Randomized Controlled Trial"[pt] OR "Controlled Clinical Trial"[pt]) Sensitivity search (study-design filter omitted): (("Osteonecrosis of the Jaw"[Mesh] OR "Bisphosphonate-Associated Osteonecrosis of the Jaw"[Mesh] OR "medication-related osteonecrosis of the jaw"[tiab] OR MRONJ[tiab] OR "bisphosphonate-related osteonecrosis of the jaw"[tiab] OR BRONJ[tiab] OR "osteonecrosis of the jaw"[tiab]) AND ("Laser Therapy"[Mesh] OR laser*[tiab] OR "Er:YAG"[tiab] OR erbium[tiab] OR "Nd:YAG"[tiab] OR "CO2 laser"[tiab] OR "carbon dioxide laser"[tiab] OR diode[tiab] OR photobiomodulation[tiab] OR "low-level laser"[tiab]) AND (surg*[tiab] OR debridement[tiab] OR curettage[tiab] OR sequestrectomy[tiab] OR treatment[tiab]))	No date restriction.
Embase	Primary RCT-focused search (Emtree/free text): ('jaw osteonecrosis'/exp OR 'medication related osteonecrosis of the jaw':ti,ab,kw OR MRONJ:ti,ab,kw OR 'bisphosphonate related osteonecrosis of the jaw':ti,ab,kw OR BRONJ:ti,ab,kw OR 'osteonecrosis of the jaw':ti,ab,kw) AND ('laser therapy'/exp OR laser*:ti,ab,kw OR 'Er:YAG':ti,ab,kw OR erbium:ti,ab,kw OR 'Nd:YAG':ti,ab,kw OR 'CO2 laser':ti,ab,kw OR diode:ti,ab,kw OR photobiomodulation:ti,ab,kw OR 'low level laser':ti,ab,kw) AND (surg*:ti,ab,kw OR debridement:ti,ab,kw OR curettage:ti,ab,kw OR sequestrectomy:ti,ab,kw OR treatment:ti,ab,kw) AND (random*:ti,ab,kw OR trial:ti,ab,kw OR controlled:ti,ab,kw OR 'randomized controlled trial'/exp) Sensitivity search: same disease, laser, and surgical-treatment concepts with the study-design block omitted.	Emtree plus title/abstract/keyword terms; no date restriction.
Scopus	Primary RCT-focused search: TITLE-ABS-KEY(("medication-related osteonecrosis of the jaw" OR MRONJ OR "bisphosphonate-related osteonecrosis of the jaw" OR BRONJ OR "osteonecrosis of the jaw") AND (laser* OR "Er:YAG" OR erbium OR "Nd:YAG" OR "CO2 laser" OR "carbon dioxide laser" OR diode OR photobiomodulation OR "low-level laser") AND (surg* OR debridement OR curettage OR sequestrectomy OR treatment)) AND TITLE-ABS-KEY(random* OR trial OR controlled) Sensitivity search (study-design filter omitted): TITLE-ABS-KEY(("medication-related osteonecrosis of the jaw" OR MRONJ OR "bisphosphonate-related osteonecrosis of the jaw" OR BRONJ OR "osteonecrosis of the jaw") AND (laser* OR "Er:YAG" OR erbium OR "Nd:YAG" OR "CO2 laser" OR "carbon dioxide laser" OR diode OR photobiomodulation OR "low-level laser") AND (surg* OR debridement OR curettage OR sequestrectomy OR treatment))	TITLE-ABS-KEY search; no date restriction.
Cochrane Library (CENTRAL)	([mh "Osteonecrosis of the Jaw"] OR [mh "Bisphosphonate-Associated Osteonecrosis of the Jaw"] OR "medication-related osteonecrosis of the jaw":ti,ab,kw OR MRONJ:ti,ab,kw OR "bisphosphonate-related osteonecrosis of the jaw":ti,ab,kw OR BRONJ:ti,ab,kw OR "osteonecrosis of the jaw":ti,ab,kw) AND ([mh "Laser Therapy"] OR laser*:ti,ab,kw OR "Er:YAG":ti,ab,kw OR erbium:ti,ab,kw OR "Nd:YAG":ti,ab,kw OR "CO2 laser":ti,ab,kw OR diode:ti,ab,kw OR photobiomodulation:ti,ab,kw OR "low-level laser":ti,ab,kw) AND (surg*:ti,ab,kw OR debridement:ti,ab,kw OR curettage:ti,ab,kw OR sequestrectomy:ti,ab,kw OR treatment:ti,ab,kw)	CENTRAL is trial-focused; no separate RCT filter required.

Table 5 lists the outcome-level RoB 2 rationale.

**Table 5:** Outcome-level Cochrane RoB 2 rationale

Domain	Judgement	Rationale	Outcomes affected
D1. Randomisation process	Low risk	Publication reports software-generated random allocation with sealed-envelope concealment. The inconsistent participant counts are important reporting concerns but do not by themselves demonstrate a nonrandom sequence or compromised concealment.	Healing, complications, necrosis, pain, duration
D2. Deviations from intended interventions	Low risk	No material crossovers or deviations from assigned treatment were reported. The operating surgeon was necessarily unblinded, but this alone does not indicate deviations from intended intervention.	All assessed outcomes
D3. Missing outcome data	Some concerns	The Methods/Results describe 72 randomised but only 65 completing/analyzed. Missingness was unbalanced (6 conventional vs 1 laser). Reasons included cancer deterioration and relocation, so a relationship between missingness and outcome cannot be excluded.	All assessed outcomes
D4. Measurement of outcome	Some concerns	Publication reports that patients, the postoperative assessor, and the data analyst were blinded while the surgeon was not; ClinicalTrials.gov [23] lists no masking. Healing, complications, necrosis, and pain therefore retain uncertainty in outcome measurement. Procedure duration is objectively timed.	Healing, complications, necrosis, pain
D5. Selection of reported result	Some concerns	Trial registration was retrospective. Participant counts differ between the abstract, Methods/Results, and registry, and some outcome timing or definitions are incompletely specified, raising reporting concerns.	All assessed outcomes
Overall	Some concerns	No domain was judged high risk, but missing outcome data and reporting/measurement uncertainties prevent a low-risk overall judgement.	All assessed outcomes

Table 6 lists the review-calculated effect estimates.

**Table 6:** Review-calculated effect estimates Risk ratios were calculated with the Wald log-risk-ratio method from published event counts. Mean-difference confidence intervals use a normal-approximation two-sample standard error from reported means, standard deviations, and group sizes. These are single-study calculations, not pooled meta-analytic estimates.

Outcome	Laser	Conventional	Measure	Estimate (95% CI)	Interpretive note
Complete mucosal healing	30/35	8/30	RR	3.21 (1.75-5.91)	RR >1 favors laser-assisted surgery.
Postoperative complications	6/35	18/30	RR	0.29 (0.13-0.63)	RR <1 favors laser-assisted surgery.
Grade II/III necrosis at 3 months	1/35	10/30	RR	0.09 (0.01-0.63)	Sparse-event estimate; magnitude is unstable.
Pain at 3 months	1.34 ± 1.39 (n=35)	3.93 ± 2.68 (n=30)	MD	-2.59 (-3.65 to -1.53)	Negative MD favors laser-assisted surgery.
Pain at 6 months	0.60 ± 0.95 (n=35)	3.13 ± 2.52 (n=30)	MD	-2.53 (-3.49 to -1.57)	Negative MD favors laser-assisted surgery.
Procedure duration, min	70.03 ± 5.95	57.90 ± 7.84	MD	+12.13 (8.70 to 15.56) min	Laser-assisted surgery took longer; CI calculated by review authors from reported means, SDs, and group sizes.

Table 7 provides the extreme-case sensitivity analysis for missing healing outcomes.

**Table 7:** Extreme-case sensitivity analysis for missing healing outcomes

Scenario	Laser healed	Conventional healed	RR (95% CI)	Interpretation
Published complete-case analysis	30/35	8/30	3.21 (1.75-5.91)	Primary review calculation.
Most favorable to laser	31/36	8/36	3.88 (2.07-7.24)	Missing laser patient assumed healed; all six missing conventional patients assumed unhealed.
Least favorable to laser	30/36	14/36	2.14 (1.39-3.31)	Missing laser patient assumed unhealed; all six missing conventional patients assumed healed.

Table 8 lists the GRADE summary of findings.

**Table 8:** GRADE summary of findings and certainty of evidence GRADE: Grading of Recommendations Assessment, Development and Evaluation; RR: risk ratio; MD: mean difference; CI: confidence interval; RCT: randomised controlled trial; VAS: visual analog scale. GRADE was added after protocol registration and is therefore exploratory [26]. Randomised evidence began at high certainty. No downgrade was applied for inconsistency or publication bias solely because only one study was available; these domains could not be assessed empirically. No serious indirectness was judged when the evidence is interpreted specifically as adjunctive Er:YAG surgery for stage II-III MRONJ. Findings should not be generalized to other laser modalities or disease stages. Reasons for downgrading are shown for each outcome above.

Outcome	Effect estimate	Participants	GRADE certainty	Reason for rating
Complete mucosal healing	RR 3.21 (95% CI 1.75-5.91); 85.7% vs 26.7%	65 (1 RCT)	Low	Low certainty: downgraded one level for risk of bias (unbalanced incomplete outcome data and reporting inconsistencies) and one for imprecision because the estimate comes from a single small trial although the CI excludes the null.
Postoperative complications	RR 0.29 (95% CI 0.13-0.63); 17.1% vs 60.0%	65 (1 RCT)	Low	Low certainty: downgraded one level for risk of bias and one for imprecision. Only 24 complication events were observed in one small trial, although the CI excludes the null.
Grade II/III necrosis at 3 months	RR 0.09 (95% CI 0.01-0.63); 2.9% vs 33.3%	65; 11 events (1 RCT)	Very low	Very low certainty: downgraded one level for risk of bias and two for very serious imprecision. Only 11 events occurred, including one event in the laser group, making the magnitude of effect unstable.
Pain at 3 months	MD -2.59 (95% CI -3.65 to -1.53)	65 (1 RCT)	Low	Low certainty: downgraded one level for risk of bias and one for imprecision. The trial is small, the registry-publication masking discrepancy affects a patient-reported outcome, and no minimally important difference was prespecified by the review.
Pain at 6 months	MD -2.53 (95% CI -3.49 to -1.57)	65 (1 RCT)	Low	Low certainty: downgraded one level for risk of bias and one for imprecision for the same reasons as the 3-month pain outcome.
Procedure duration	MD +12.13 min (95% CI 8.70-15.56)	65 (1 RCT)	Moderate	Moderate certainty: downgraded one level for risk of bias/reporting concerns. Duration is objectively timed and the CI around the observed difference is comparatively narrow.

Appendix B

Appendix B includes the standardised data-extraction fields, key near-eligible exclusion rationale, and source-verification notes for the included trial.

S1. Standardised Data-Extraction Fields

·       Study identification: first author, year, country, center(s), publication type, registry identifier.

·       Design: randomised sequence-generation method, allocation concealment, masking, prospective/retrospective registration, funding, conflicts of interest.

·       Population: diagnostic definition, MRONJ/BRONJ stage, medication exposure, age, sex, relevant comorbidity information, number assessed, randomised, analysed, and lost to follow-up.

·       Intervention: laser modality, wavelength/device, energy, power, frequency, pulse mode, tip diameter, cooling, concomitant debridement and perioperative therapy.

·       Comparator: conventional surgical method, instrumentation, cooling/irrigation, concomitant perioperative therapy.

·       Outcomes: complete mucosal healing, postoperative complications, grade II/III necrosis, pain, procedure duration, definitions and assessment time points.

·       Numerical data: events and denominators for dichotomous outcomes; means, standard deviations, and group sizes for continuous outcomes; reported effect estimates and P values.

·       Risk of bias: RoB 2 signalling questions and domain-level/overall judgements by outcome.

·       Notes: discrepancies between publication, registry, protocol/SAP, and bibliographic indexing.

S2. Key Near-Eligible Exclusion Rationale

**Table 9:** Key near-eligible full-text exclusion requiring explicit justification

Study	Reason for exclusion from randomised evidence base	Context
Atalay et al. (2011) [22]	Excluded from the primary randomised evidence base because the prespecified eligibility criterion required a documented random sequence-generation method. The abstract describes a retrospective study; the Methods state that patients were 'randomly divided' but do not report how the sequence was generated or concealed.	Near-eligible comparative evidence: 20 stage I-II BRONJ patients; Er:YAG surgery plus postoperative Nd:YAG biostimulation versus conventional surgery; complete healing 70% vs 40%, P = .370. The design description is internally inconsistent.

S3. Source-Verification Notes for the Included Trial

The Michalak et al. [15] publication contains inconsistent participant accounting. The abstract reports 65 patients randomly allocated (35 laser-assisted; 30 conventional), while the Methods state that 106 patients were assessed and 72 were allocated (36 per group) using a software-generated random table with sealed-envelope concealment. The Results again state that 72 were randomised and 65 completed follow-up. The report states that 41 of 106 assessed patients were excluded, which would leave 65 rather than 72. ClinicalTrials.gov [23] reports actual enrollment of 65. Effect estimates in this review therefore use the 65 participants with published outcome data; the seven additional participants described in the Methods/Results are addressed only in the missing-data sensitivity analysis.

The publication describes the operating surgeon as unblinded and the patients, postoperative outcome assessor, and data analyst as blinded. The registry lists no masking. This discrepancy is reflected in the RoB 2 measurement and reporting judgements rather than being resolved by assumption.
